# Occlusal Analysis in Natural Dentition: Systematic Review

**DOI:** 10.1055/s-0042-1755626

**Published:** 2022-10-17

**Authors:** Byron Velásquez, María Rodríguez, Verónica Mosquera, Eddy Álvarez, Luis Chauca, Alexandra Mena

**Affiliations:** 1 Department of Prosthesis, School of Dentistry, Universidad de Las Americas, Quito, Ecuador; 2 Department of Prosthesis, School of Dentistry, Universidad de Las Americas, Quito, Ecuador; 3 Department of Periodontics and Implantology Oral Research, College of Dentistry, Universidad de Guayaquil, Guayaquil, Ecuador; 4 Departamento de Investigación de Prótesis Dental, Facultad de Odontología, Universidad de Las Américas, UDLACP, Quito, Ecuador

**Keywords:** dental occlusion, occlusal adjustment, temporomandibular joint, mastication, bite force

## Abstract

This study aimed to evaluate the effectiveness of conventional occlusal analysis in contrast with digital occlusal analysis in natural dentition. Occlusal analysis allows the identification of normal and abnormal occlusal contact points that alter the craniomandibular cervical system. We searched for articles with keywords [[dental occlusion]], [[natural dentition]], [[occlusal adjustment]], [[Immediate Complete Anterior Guidance Development]] [[mastication]], [[bite force]], [[premature contact]], [[occlusal balance]] [[articulating paper]]], [[spray]], [[Occlusal contacts]], and [[bite strength]]. They were considered observational , odds ratio and case control studies. We found 189 items. After evaluating the abstracts and full texts of the articles, 10 papers met the inclusion criteria. It was found that occlusal analysis allows the identification of the relationship between poor occlusion and the sensitivity of the teeth due to occlusal trauma, which is also related to temporomandibular joint pain in dynamic occlusion. The contacts of greater strength were observed in nonfunctional cusps, 48%, without ruling out the functional cusps, 24%. Despite being the universal method of occlusal control to date, the use of joint paper, remains subjective compared to the digital occlusal control device. Posture is considered directly related to occlusal trauma and temporomandibular disorders; without proper occlusal analysis, a clear diagnosis of the patient's joint condition cannot be obtained. Digital occlusal analysis is more objective than traditional occlusal analysis.

## Introduction

Occlusion is morpho-physiologically static, dynamic, and uniaxial and can be altered by modifications in the stomatognathic system, teeth, muscles, ligaments, and temporomandibular joint. 1 2 Occlusive analysis allows the identification of normal and abnormal occlusal contact points. In occlusion, marks are used to indicate the location of contact points after asking the patient to make mandibular movements such as opening, closing, lateral movements (right and left), protrusion, and retrusion. 2 Marks can be made by spray or paper. The fidelity of these systems depends on the operator's ability, the sensitivity of the marker, etc. 3 4 When thinner articulating paper is used, unnecessary wear on teeth can be prevented. Additionally, some commercial brands do not adhere to the thickness guidelines of occlusive paper. 5 6 Computerized digital occlusal analysis appears to be a more precise method; it uses technological benefits to achieve the correct occlusal diagnosis, measure relative occlusal forces in static and dynamic mandibular positions, and generate three-dimensional graphics that identify the areas of greater or less pressure. 7 Despite the advantages of the digital method, articulating paper is the most used method by clinical dentists. By varying occlusal effects in endodontic teeth and utilizing digital or traditional occlusal analysis, 8 we determined that occlusal contact can be reduced with occlusal adjustment. A similar analysis of occlusal force is measured in overdentures in total edentulous patients, indicating greater force measured with a digital system in the posterior sector. 9 Simultaneous occlusal contacts observed with articulating paper 10 and digital occlusal analysis 11 allow us to differentiate moments of occlusion and disocclusion, which appear visually on the teeth with the help of articulating paper, as well as in the digital occlusal analysis. 12 The aim of this study is to evaluate the effectiveness of conventional occlusal analysis in contrast with digital occlusal analysis in natural dentition.

## Methods

Investigators performed a systematic review of selected quantitative studies. The review was performed in accordance with the Preferred Reporting Items for Systematic Review and Meta-Analysis (PRISMA) statement. The review protocol was registered *a priori* , is published online in the International Prospective Register of Systematic Reviews (PROSPERO) database registration number: CRD42020146370.

The PICO question was as follows: To determine the efficacy of occlusal analysis in natural dentition with joint paper and digital occlusal analysis. Population: Patients with occlusal pathologies. Intervention: Intervention of all patients with occlusal pathologies. Comparison: Comparison of occlusal analysis in natural dentition with joint paper and computerized analysis. Outcome: Observation of mandibular crane cervix after occlusal analysis. Observational studies, case–control, odds ratio, research by keywords [[dental occlusion]], [[natural dentition]], [[Immediate Complete Anterior Guidance Development]], [[occlusal adjustment]], [[mastication]], [[ bite force]], [[premature contact]], [[occlusal balance]], [[articulating paper]]], [[spray]], [[Occlusal contacts]], [[bite strength]] using the Boolean operators AND, OR, and NOT. Scopus, EBSCO, PubMed, Medline Embase, Cochrane Library, and Web Science and alternate databases Scielo, Latindex, and Redalyc were searched. Preferred Reporting Items for Systematic Review and MetaAnaysis Protocols (PRISMA) research protocol, fluxogram that explains the sequence of information selection.

**Fig. 1:**
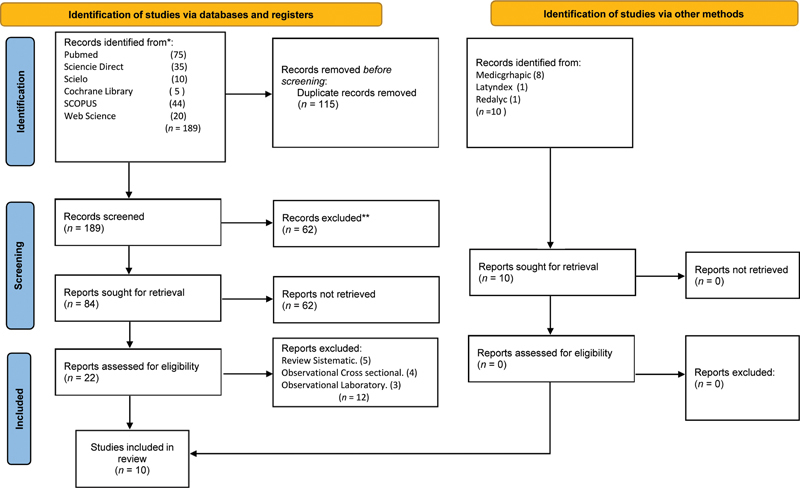
Selection Studies flow diagram.

Inclusion criteria were as follows: complete articles, dates of the articles, odds ratio, case–control with confidence interval (CI) lower and CI upper, and articles containing studies of occlusal pathology. In total, the keyword search returned *n* = 189 articles from the database and additional records identified from other sources were *n* = 10. Some articles were removed after they were found to be duplicate ( *n* = 115 records), and some were excluded for not presenting their information on occlusal pathologies, anterior guide, occlusal trauma, or papers that related to bad occlusion with bruxism or temporomandibular disorders. Articles were narrowed down to 84 after screening and excluded 62 of these so that the total number of studies included in the qualitative synthesis was 22. The total number of studies included in the final analysis was 10 ( Fig. 1 ).

This systematic review was carried out independently by two researchers different from the main one, specialists in the area of oral rehabilitation. Disagreements about study selection were resolved by consensus or intervention of the main researcher. Agreement of the selected articles by the two authors was calculated using kappa coefficient, which was 0.85. In all cases of concordance assessment, the response options were dichotomized to two response options: inclusion and exclusion. As a result, 115 articles were excluded. Of these remaining 84 articles, complete text could be not accessed in 62 articles, only the summaries, and the remaining 22 were completely reviewed by the principal investigator, excluding 25 articles for not meeting the selection criteria due to not adequately describing the clinical criteria of the diagnosis. A final total of 10 articles were obtained.

The authors (B.V.V.R., L.C.H.B., M.R.T., and V.M.C.) independently selected the titles and summaries, excluded duplicates and irrelevant articles, and considered only full-text articles. The date and names of all authors were included in the final review article. Any conflict with respect to inclusion and exclusion criteria was resolved by the third and fourth authors (A.M.S., E.A.). The data extraction procedure was evaluated according to the criteria of all authors. Articles are classified by the author/year, study objective, study type, methodology, the results (standard mean and deviation), and conclusions.

## Results

To analyze the research articles, the risk of bias was assessed. Bias is assessed as a judgment for individual elements from domains (selection, reporting, and other) ( Table 1 ). When assessing to clarify the gold standard, the collection of information was not homogeneous between the studies, and none reported having considered the reliability and validity of the clinical aspects. The classification system had a dental evaluation to classify patients and was able to apply conventional occlusal analysis or digital analysis. Methods varied between questionnaires (with dichotomous yes/no, multiple-choice, or visual analog scale responses), interviews, the use of immediate complete anterior guidance development, and digital occlusal analysis ( Table 2 ). Acceptable intra- and interexaminer reliability considered the previous training of examiners prior to clinical measurements; in many of the studies, the number of examiners was unclear and appeared to vary between one, two, or more. The use of adequate samples varied widely in the number of subjects, although a good number of the reports included more than 30 participants. Samples also varied in age ranges, and some of the studies included patients younger than 30 years, while others included patients over 50 years. When assessing replicability of the studies, the studies that presented similar conclusions did not necessarily obey a replicability of the responses of the patients examined in the different studies, which was due to the heterogeneity of the designs and other methodological shortcomings. Without an appropriate occlusal analysis, a clear diagnosis of the patient's joint condition cannot be obtained ( Table 3 ).

**Table 1:** Summary of the risk of bias according to Consolidated Standards of Reporting Trials

Item	Arslan et al 4 2017	Basson et al 5 2020	Brizuela-Velasco et al 6 2015	Gupta et al 11 2019	Kerstein and Radke et al 14 2017	Kerstein and Radke et al 15 2012	Kerstein and Radke et al 16 2014	Kerstein 17 2008	Prafulla et al 28 2020	Yiannios et al 48 2017
1 Abstract	Yes	Yes	Yes	Yes	Yes	Yes	Yes	Yes	Yes	Yes
2a Background and objectives	Yes	Yes	Yes	Yes	Yes	Yes	Yes	Yes	Yes	Yes
2b Background and objectives	Yes	Yes	Yes	Yes	Yes	Yes	Yes	Yes	Yes	Yes
3 Intervention	Yes	Yes	Yes	Yes	Yes	Yes	Yes	Yes	Yes	Yes
4 Outcomes	Yes	Yes	No	No	Yes	No	Yes	Yes	Yes	Yes
5 Sample size	No	No	Yes	No	Yes	No	Yes	No	Yes	Yes
6 Randomization: sequence generation	No	No	No	No	No	No	No	No	No	No
7 Allocation concealment mechanism	No	No	No	No	No	No	No	No	No	No
8 Implementation	No	No	No	No	No	No	No	No	No	No
9 Blinding	No	No	No	No	No	No	No	No	No	No
10 Statistical methods	Yes	Yes	Yes	No	Yes	Yes	Yes	Yes	Yes	Yes
11 Results: outcomes and estimation	Yes	Yes	No	Yes	Yes	Yes	Yes	Yes	Yes	Yes
12 Discussion: limitations	Yes	No	Yes	Yes	Yes	Yes	Yes	Yes	Yes	Yes
13 Other information: funding	No	No	No	Yes	Yes	Yes	Yes	No	No	No
14 Protocol	Yes	No	Yes	Yes	Yes	Yes	Yes	Yes	Yes	Yes

**Table 2:** Summary of the risk of bias

Item	Arslan et al 4 2017	Kerstein and Radke et al 14 2017	Prafulla et al 28 2020	Yiannios et al 48 2017	Gupta et al 11 2019
Random sequence generation	Low	Low	Low	Low	Unclear
Allocation concealment	Low	Low	Low	Unclear	Unclear
Selective reporting	Low	Low	Low	Low	Low
Blinding (participants and personnel)	High	high	high	High	High
Blinding (outcome assessment)	High	High	High	High	High
Incomplete outcome data	Low	Low	Low	Low	Low

**Table 3:** Structural summary of systematic review

Study (year)	Object of research	Intervention	Evaluation methods	Result
Arslan et al 4 2017	Effect of various occlusal reduction levels	104 dental students	Immediate complete anterior guidance development (ICAGD) coronoplasty	Occlusal reduction ( *p* > 0.005)
Basson et al 5 2020	Visually select the 2 highest force occlusal contacts	2 choices as being the highest force 9 different contact areas	T-Scan	12 correct highest force (4.8% correct; 95.2% incorrect). 8 1st choices were correct (6.5% correct; 93.5% incorrect), 4 2nd choices were correct (3.2% correct; 96.7% incorrect)
Brizuela-Velasco et al 6 2015	Occlusal contact surface registered with an articulating paper	15 patients	Articulating paper to obtain their occlusal registrations	Articulating papers (12-pm or 40-pm) can avoid unnecessary grinding on teeth during occlusal adjustment
Gupta et al 11 2019	Compare the occlusal contacts in dentate and edentulous patients	15 dentate and 15 edentulous	T-Scan III	Area was found in dentate and denture wearers, i.e., tooth contact area varies with head posture
Kerstein and Radke et al 14 2017	To determine if reducing long disocclusion time	29 muscularly symptomatic	Immediate complete anterior guidance development (ICAGD) coronoplasty	ICAGD time reductions (2.11–0.45 s; *p =* 0.0000) muscle changes ( *p =* 0.000001) peak amplitude ( *p =* 0.00005) peak contraction ( *p <* 0.000004 50% peak contraction ( *p <* 0.00001) silent periods per side (right *p <* 0.0000002 left *p <* 0.0000006) centric occlusion ( *p <* 0.002) chewing velocities increased ( *p <* 0.002; *p <* 0.00005) opening and closing time: decreased ( *p* < 0.004–0.0001)
Kerstein and Radke et al 15 2012	Occlusal adjustment procedure, immediate complete anterior guidance developed (ICAGD)	45 chronic myalgic temporomandibular disorder (TMD) patients.	Medical history and clinical examination. Joint vibration analysis	Disocclusion time to less than 0.4 second ( *p <* 0.00014) after Bonferroni correction ( *p* < 0.0006)
Kerstein and Radke et al 16 2014	Subjective interpretation of paper marking are a reliable method	295 clinicians selected	T-Scan, articulating paper to obtain their occlusal registrations	295 dentists only chose 12.5–13.3% correct contacts by looking at the paper marks ( *p <* 0.05)
Kerstein 17 2008	Performance of a new design of occlusal sensor, high dentition (HD) sensor	40 maxillary casts with a known arch half force	Computer-guided refinement occlusal adjustments (T-Scan III)	20% reduction in mean jerkiness during opening ( *p =* 0.0002), 19% reduction in mean jerk during closing ( *p =* 0.0114) were found after ICAGD
Prafulla et al 28 2020	Occlusal adjustment study using a randomized clinical trial (RCT)	104 first year dental students	Immediate complete anterior guidance development (ICAGD) coronoplasty	1 week retest ( *p <* 0.05) Pretreatment through 6 months ( *p >* 0.05) 1 week after ICAGD ( *p <* 0.00001) 3 months retest ( *p <* 0.05)
Yiannios et al 48 2017	After subjects undergo the immediate complete anterior guidance development (ICAGD) computer-guided occlusal adjustment	100 chronically dysfunctional patients	Immediate complete anterior guidance development (ICAGD) coronoplasty spectrophotometer	Discussion time reductions (2.11–0.55 s; *p =* 0.0000)

## Discussion

Various methods and materials found in the literature that have been used for occlusal analysis have been described. 13 However, more work is needed in this field by developing new devices or improving them to ensure accurate diagnostic methods that minimize the possibility of error. 14

Qualitative analysis devices are used for clinical detection at the location of the contact point in its exact position 15 ; this type of material has the advantage of presenting a low cost and simple application, such as static occlusal devices. 16 17

With quantitative analysis devices such as digital systems dynamics, 18 19 the exact intensity has to be registered in the time of contact; however, this system does not locate the position of the contact point, as the sensor is based on standardized estimates of dental positions. However, with the new software updates 20 ^,^ 21 and the latest digital occlusal system, the individualization of the arcade with greater precision is possible. 22 For all these reasons, it is now necessary to use the combination of both systems, qualitative and quantitative 23 24 ^,^ as is the case with the role of articulation in conjunction with digital occlusal systems.

Malta et al compared the sensibility and coincidence of some studies 25 between several dispositive qualitative and quantitative studies, concluding that there are differences in the number of contacts between the three systems. 26 Both studies found a lower sensitivity of the digital system T-S which can be compared to the other systems. 27 28 Sensitivity is adversely affected when the sensors are used several times, while the role of articulation is realized at once. 29 30 In the other systems, it was observed that the number of registered contacts increased when there was no saliva on the occlusal surfaces, while this did not seem to affect the digital occlusal system. 31 32 One of the limitations of the type of device that uses sensor, 33 refers to when the thickness of the film of the device 34 is greater than it should be, the results that are obtained present older contacts of posterior teeth than in previous teeth. 35 36

Song et al demonstrated that the appropriate thickness of an occlusive is that of a joint paper to detect previous and subsequent anterior and posterior contacts 37 but without altering the patient's proprioception when biting. Thus, it has to be equal to or less than 20 μm. 35 A reviewed index assessed the reproducibility and usefulness of instruments that valued occlusion, 35 38 among them were both static and dynamic devices. It was observed how electromyographic records 39 could measure the activation of the jaw musculature and its position, although the cost–benefit relationship of this type of device is not clear. 40 Finally, the authors concluded that none of the instruments reviewed in the different publications presented results that validated their use and reproducibility. 41 42

Some authors recommend that the collection of static and dynamic occlusal data 43 did not have reliability by the operator, resulting in problems in the design study 44 and the lack of standardization.

The most common indicators of static occlusion used by dentists are role of paper articulating, 45 seda, and nylon, in clinical practice 46 ^,^ which allows subjectivity at the moment of occlusal analysis, even if the mark of the indicators is considered and similarity in the variants between the digital systems ( Table 4 ). Variations in the mark may result in differences among observers (dentists), 47 while the clinician may remove tooth structure during an occlusal adjustment, another may not see it as convenient. The clinical judgment will depend not only on the knowledge about the occlusion of the individual himself but also on the mark that the indicator used. 48

**Table 4:** Occlusal indicators

System	Thickness	Localization	Force	Time
Articulating paper	20 μm	+	+	+
T-Scan system	60 μm	+	+	−
Prescale dental	98 μm	+	−	−

The occlusion is not static but has dynamism; consequently, it must be recorded 49 and the information obtained from the static indicators for the analysis of the occlusal contacts should be complemented with that of dynamic indicators such as digital occlusal systems. 50 51

## Conclusion

Digital occlusal analysis in most interventions is more objective than traditional occlusal analysis.

## Author Contribution

**Table:** 

1. Conceptualitation	B.V.R.
2. Data curation	A.M.S./E.A.L.
3. Formal Analysis	B.V.R., V.M.C.
	L.C.B., M.R.T.
4. Acquicition of funds	B.V.R., A.M.S.
5. Research:	B.V.R., V.M.C.,
	L.C.B., M.R.T.
	E.A., A.M.S.
6. Methodology	B.V.R., A.M.S.
7. Proyect administration	B.V.R.
8. Means	V.M.C., L.C.B., M.R.T., E.A.
9. Software	A.M.S.
10. Supervición	B.V.R., A.M.S.
11. Validation	B.V.R., V.M.C.
	L.C.B., M.R.T.
	E.A., A.M.S.
12. Visualitation	B.V.R., A.M.S.
13. Drafting – original draft	B.V.R., V.M.C.
	L.C.B., M.R.T.
	E.A., A.M.S.
14. Drafting – review and edition	All the authors

## Clinical Relevance

This study tries to remind professionals of the importance of occlusal analysis considering it a mandatory diagnostic means in any multidisciplinary preclinical process, determining that digital occlusal analysis limits the subjectivity of conventional occlusal analysis.
